# Psychometric Properties of the Sexual Interest and Desire Inventory-Female for Diagnosis of Hypoactive Sexual Desire Disorder: The Persian Version

**Published:** 2016-10

**Authors:** Mina Malary, Mehdi Pourasghar, Soghra Khani, Mahmood Moosazadeh, Zeinab Hamzehgardeshi

**Affiliations:** 1 Department of Reproductive Health and Midwifery, Mazandaran University of Medical Sciences, Sari, Iran.; 2 Student Research Committee, Mazandaran University of Medical Sciences, Sari, Iran.; 3 Department of Psychiatry and Research Center for Psychiatry and Behavioral Sciences, Mazandaran University of Medical Sciences, Sari, Iran.; 4 Sexual and Reproductive Health Research Center, Mazandaran University of Medical Sciences, Sari, Iran.; 5 Research Center of Diabetes, Mazandaran University of Medical Sciences, Sari, Iran.; 6 Health Sciences Research Center, Faculty of Health, Mazandaran University of Medical Sciences, Sari, Iran.; 7 Traditional and Complementary Medicine Research Center, Mazandaran University of Medical Sciences, Sari, Iran.

**Keywords:** *Hypoactive Sexual Desire Disorder*, *Sexual Health Interest and Desire Inventory-Female*, *Psychometric*

## Abstract

**Objective:** Hypoactive sexual desire Disorder (HSDD) is a common sexual problem among women. Sexual interest and desire inventory –female (SIDI-F) has been widely validated and used to measure sexual desire in women. The aim of this study was to determine the psychometric properties of the Persian version of SIDI-F for Iranian population.

**Method:** This was a methodological study on the psychometric properties of SIDI –F. This report describes the process and principles used in the translation and cultural adaptation of the SIDI-F on 40 women of reproductive age who were selected using convenience sampling method. The content validity of this inventory was proved by analyzing the feedback solicited from women of reproductive age; professions specialized in health, sociology and psychology. Reliability was assessed through test-retest and internal consistency reliability.

**Results:** Few cultural differences were identified and considered during the process of translation and validation. In Content Validity Ratio (CVR) measurement, the total score of SIDI-F was higher than Lawsche table (%51 for 14 experts), indicating the importance of including the mentioned items in the tool. CVR scores for all items were equal or more than 0.79. The internal consistency reliability measured for the whole tool was 0.89, showing considerable total reliability.

**Conclusion**: The Persian version of the SIDI-F seems to be valid and reliable and can be used to identify women with low sexual desire through research and sexual health programs provided by the health centers in Iran, and to design appropriate interventions to treat HSDD.

It is believed that low or decreased sexual desire that causes personal distress is the core symptom of hypoactive sexual desire disorder (HSDD) (1). 

According to the American Psychiatric Association’s Diagnostic and Statistical Manual IV-TR (DSM-IV-TR), HSDD is defined as the recurrent or permanent decrease or lack of sexual desire and fantasy for conducting sexual activity which can cause interpersonal discomfort and problems (2(. The prevalence of HSDD varies from 5 to 55%, depending on the sample population under evaluation, the instrument used for assessment and the diagnostic criteria applied (3, 4). In a big study on Brazilian women, the prevalence of HSDD was reported to be 9.5% (5). In a systematic review and meta-analysis study conducted in Iran, the prevalence of HSDD was estimated to be 35% in the general population (6). 

Lack or decrease of sexual desire indicates a serious problem which brings major consequences on women's life quality, feeling of being healthy and interpersonal relationships. Women with HSDD may report little or no interest in having a sexual relationship, inability to respond to eroticism or feeling numb despite having a good relationship with their partners (7). Despite the negative effects that low desire may have on life quality of these women, many are reluctant to speak about their sexual issues with doctors, and many doctors do not feel comfortable answering sexual complaints (8). It is difficult to measure sexual desire because it has a multi-dimensional structure with biological, cognitive and emotional aspects (9). Furthermore, sexual desire and behavior are not completely related to each other, so it has been shown that women may take part in sexual activities without having desire for it, or may or may not participate in sexual activities for a reason not related to sexual desire (10). Introducing simple assessment tools encourages doctors to discuss sexual issues as a part of patients’ normal encounter and helps doctors and patients to feel more comfortable discussing this issue. 

Although some tools have been developed to assess overall sexual function (female sexual function index (FSFI) (11), and changes in sexual function questionnaire- female (CSFQ -F) (12)), and specially low sexual desire (sexual desire inventory, sexual desire and arousal inventory, Halbert 's sexual desire index, and signs for scaling sexual desire) (13-16)), no assessment has been done on different aspects of this specific disorder, and no structured approach is available for the doctors to assess HSDD. Therefore, sexual interest and desire inventory-female has been developed by Clayton et al. (2006) as a clinician- administered instrument to assess the intensity of HSDD and as a complete interview for a comprehensive understanding of sexual desire (17). 

The aim of this study was to determine the psychometric properties of sexual interest and desire inventory-female, the Persian version, to be used for the Iranian population.

## Materials and Method

This was a methodological study of psychometric properties of SIDI –F. This report describes the process and principles used in the translation and cultural adaptation of the SIDI –F to ensure the validity and reliability of the tool. In the summer of 2015, forty Iranian married women of reproductive age (15-49 yrs.) in the city of Sari with at least 6 months married life who were willing to participate in this assessment were selected through convenience sampling method. Those who were pregnant or in their first six- month of breastfeeding and those afflicted by premature menopause were excluded.

In the first and second assessments, 40 women of reproductive age (29.85±7.22) filled in the questionnaires completely. The education level of the participants ranged from having a primary school education to a university degree, and the response rate to the questionnaire was 100%. The SIDI-F questionnaire is a clinician-rated inventory, which contains 13 items (relationship-sexual, receptivity, initiation, desire- frequency, affection, desire satisfaction, desire-distress, thoughts-positive, erotica, arousal-frequency, arousal ease, arousal continuation and orgasm) plus five diagnostic modules. Diagnostic module items are common interfering conditions (including relationship – general, thoughts – negative, fatigue, depression, pain) and their scores are not included in the total score. SIDI-F questionnaire used two types of scoring: 8 items are scored only by the intensity of the symptoms and 5 items are scored both by intensity and frequency of the symptoms. Each item can get a score of 0, 1, 2, 3, 4 or 5- depending on the item. The total score falls within 0 to 51, and a higher score indicates better sexual function, so in screening the women, the score of 33 and lower indicates the existence of HSDD and a higher score indicates the lack of HSDD (8).

Ethical approval for the study was obtained from The Ethics Committee of Mazandaran University of Medical Sciences. First, approval to translate and use the SIDI-F questionnaire was obtained from its creator, Professor Clayton from Virginia University on 1/26/2015. The choice guide we used to develop our approach for the cultural adaptation and translation of SIDI-F was based on reviewing the steps of International Quality Of Life Association (IQOLA) (18). The inventory was translated into Farsi by two Persian translators. The review and comparison of the two scripts were done by both translators and the head researcher. The final version of the questionnaire was agreed upon after selecting the most appropriate translated phrases, and then two English translators translated the script into English again to ensure the accuracy of the primary translation. One of the two translators was a native English speaker and had no knowledge of medicine and had never seen the original English version. Finally, the translated English version and the original checklist version were compared and the differences were discussed with the research team and translators and then the final Farsi version of the SIDI-F was prepared. 

To determine the face validity and time to complete the questionnaire, the Farsi version of SIDI-F was piloted among 40 married women with the age range of 15-49 years. The data collected from face-to-face interviews were checked to determine the participants' understanding and interpretation of the questionnaire (the words, phrases, grammar, etc.). Impact scores of the items were measured using a formula (19, 20).

Impact score equal or above 1.5 was identified as an important item.

The qualitative content validity of the Farsi version of SIDI-F was determined by 14 experts in the field of sexual and reproductive health and psychiatry (Experts who had at least 10 years of research or clinical practice on patients with sexual dysfunction). They were asked to provide their written opinion after the careful study of the inventory. Moreover, the grammar was qualitatively analyzed, and using appropriate words and patting items in the correct places were checked. With respect to the content validity, the importance and accuracy of the items were estimated using Content Validity Ratio (CVR). Moreover, to ensure that the items had been designed well, the content validity index (CVI), with a direct feedback from the panel of experts with 14 experts in the field of pregnancy health and psychiatry, was collected. CVR was measured by the formula after responding to the three choices of "the item is necessary ","the item is useful, but not necessary" and "the item is not necessary" (21). According to Lawsche table, the value of CVR for the 14 experts was considered above 51% as a necessary item in the instrument. The Farsi version of SIDI-F was given to the panel of experts again to have their opinions about how clear, simple and related each item was in a 4 score Likert scale. Then CVI was calculated using the formula (22). The criteria below were used for qualitative values of CVI: Below 0.70: Unacceptable; 0.7-0.78: Revision and correction; and equal or above 0.79: Suitable (23). 

The research team did not want to eliminate any of the items from the inventory. Therefore, items with inappropriate scores in all the three measurements of impact score, CVR and CVI were reconsidered and revision was done with the fewest changes compared to the original version. In the next step, to determine CVI, the inventory was given again to 14 experts from the previous team. This process enabled us to confirm the quantitative method of content validity as much as possible. After obtaining the new measurement of CVI from the revised items, the average for the scale of content validity index (S -CVI /Ave) was calculated according to the average score from the CVI of all items. According to the results from the pilot study and experts' opinions, necessary changes were exerted and the Farsi version of SIDI-F was properly revised. Finally, the face validity and content validity of SIDI-F were calculated and the final checklist was obtained. 

In the next step, test-retest and internal correlation were used to assess the reliability of the Farsi version of SIDI-F tool. To do so, 40 women of reproductive age in the age range of 15-49 years were selected through convenience sampling. The participating women were willing to take part in this study and completed the Farsi version of SIDI-F instrument anonymously twice with the interval of two weeks (14 days). SPSS Version 18 was used to analyze the data.

**Figure1:**
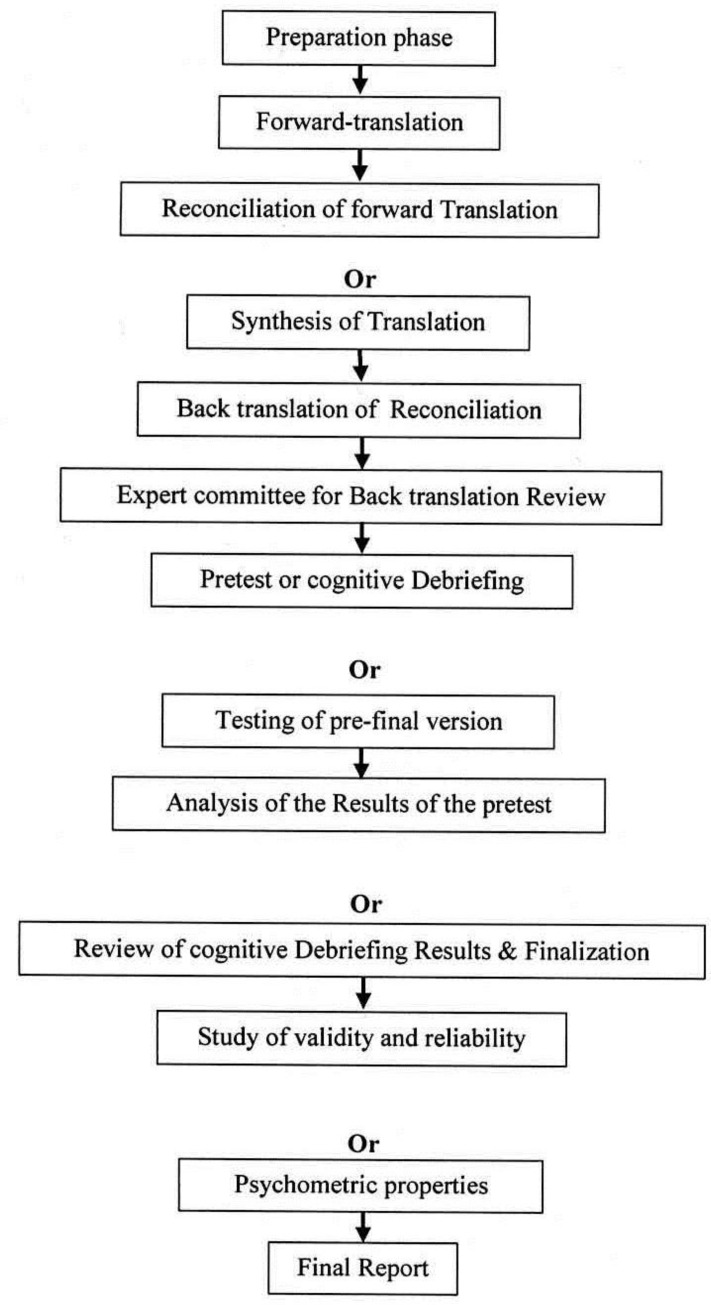
The Algorithm for the Different Steps of Translation and Cultural Adaptation

In the final phase, a report of the translation, cultural adaptability, determination of validity and reliability and the final version of the translated instrument in the written form were sent to the original makers. A summary of the steps are shown in Figure 1.

## Results

In general, the questionnaire was clear and understandable, and less than 15 minutes was enough to complete it. Results from the quantitative face validity (impact score) revealed that all items had impact scores equal or more than 1.5 (impact score ≥ 1.5), indicating the importance of these items in the target group (women of reproductive age). 

In measuring Content Validity Ratio CVR, the scores of 13 items, plus 5 diagnostic items (51%) were higher than Lawsche table (%51 for 14 experts), which indicates that the mentioned items should be included in the tool. CVR scores for all items were equal or more than 0.79 (≥0.79), so the items were identified as “necessary”. The score of only one item was between 0.7-0.79, which was reconsidered and revised (Table 1). Considering the results of the pilot phase and suggestions of the experts and the research team, we provided brief explanations for some items, and made small changes to make the items more suitable with respect to the Iranian culture. Internal consistency for the whole scale was estimated to be 0.89 using Cronbach's alpha. The reliability was obtained with the interval of two weeks, using test-retest (Table 2).

## Discussion

The psychometric properties of SIDI-F were reviewed in this study. We did not face serious problems during the process of translation and cultural adaptation. Therefore, it was not necessary to make considerable changes to the original version. We made small changes in the tool such as providing parenthesized explanations, or substituting some words and phrases in the original version of SIDI-F with more comprehensible words in Farsi. In other words, it can be stated that there was a close correlation between the English and Farsi versions of the SIDI-F.

The results revealed that SIDI-F had a satisfying reliability. The internal correlation coefficient of the tool was obtained to be 0.89 by Cronbach's alpha which is similar to the original version of the instrument (0.90) (17). Cronbach's alpha of 0.7 and higher is usually considered as a satisfactory level of internal correlation (24). Test-retest assessment with the interval of two weeks showed a proper consistency of the tool. To assess validity, we used face validity and content validity. Content validity is basically related to the understanding of text by the target group. The instrument should be understandable by the target group to encourage them to participate and respond. To ensure content validity, the content of the questionnaire was investigated. A questionnaire has content validity when its questions assess and measure all the goals of the test. Content validity was primarily assessed by the experts. 

**Table1:** Content Validity Ratio (CVR) and Content Validity Index (CVI) the Sexual Interest and Desire Inventory-Female Persian Version

	**Item**	**CVR**	**R-CVI**	**S-CVI**	**C-CVI**	**Result**
1	Relationship - Sexual	85%	85%	78%	71%	Accepted
2	Sexual Activity	71%	85%	85%	92%	Accepted
3	Receptivity	1	1	92%	92%	Accepted
4	Initiation	71%	78%	78%	78%	Accepted
5	Desire - Frequency	1	1	1	1	Accepted
6	Affection	1	1	1	1	Accepted
7	Desire - Satisfaction	85%	85%	92%	92%	Accepted
8	Desire - Distress	85%	85%	92%	92%	Accepted
9	Thoughts - Positive	1	1	1	1	Accepted
10	Erotica	1	1	1	92%	Accepted
11	Arousal - Frequency	1	1	1	1	Accepted
12	Arousal Ease	1	1	92%	78%	Accepted
13	Arousal Continuation	85%	92%	1	1	Accepted
14	Orgasm	1	1	1	1	Accepted
**Sexual Interest And Desire Inventory - Female (SIDI-F): Diagnostic Module**						
1	Relationship - General	85%	92%	1	1	Accepted
2	Thoughts - Negative	57%	85%	85%	85%	Accepted
3	Pain	85%	92%	92%	92%	Accepted
4	Mood	57%	85%	92%	85%	Accepted
5	Fatigue	52%	85%	92%	85%	Accepted

**Table2:** Reliability Index in the Dimension of Repeatability of the Sexual Interest and Desire Inventory-Female Persian Version

**Item**		**ICC** *		
		**Mean**	**Minimum**	**Maximum**
1	Relationship - Sexual	0.958	0.920	0.978
	Sexual Activity	0.989	0.978	0.994
2	Receptivity	0.957	0.920	0.977
3	Initiation	0.985	0.972	0.992
4	Desire - Frequency	0.983	0.968	0.991
5	Affection	0.971	0.945	0.985
6	Desire - Satisfaction	0.960	0.924	0.979
7	Desire - Distress	0.776	0.577	0.822
8	Thoughts - Positive	0.988	0.977	0.993
9	Erotica	0.934	0.876	0.965
10	Arousal - Frequency	0.976	0.955	0.987
11	Arousal Ease	0.980	0.963	0.990
12	Arousal Continuation	0.957	0.918	0.977
13	Orgasm	0.990	0.981	0.995
**Sexual Interest And Desire Inventory - Female (Sidi-F): Diagnostic Module**				
1	Relationship - General	0.982	0.965	0.990
2	Thoughts - Negative	0.978	0.958	0.988
3	Pain	0.844	0.708	0.918
4	Mood	0.928	0.868	0.962
5	Fatigue	0.962	0.929	0.980

* Intraclass correlation coefficient

SIDI-F questionnaire has been translated into 10 European languages, and its accuracy and usages have been proven through back-translation and linguistic validation. Clayton (2006) conducted a study on 90 women aged 18-65 years (31 women with HSDD, 24 with female orgasm disorder (FOD) and 35 without sexual disorder) from three racial backgrounds of Caucasian, African-American and Asian to assess the reliability and validity of the SIDI-F questionnaire. It was shown that this questionnaire had proper reliability and validity to assess the intensity of HSDD. In the convergent validity, the total score of SIDI-F was considerably correlated with the total scores of the two scales of female sexual function index (FSFI) and changes in sexual functioning questionnaire-female (CSFQ -F). With the combined data of the three populations, the total score of SIDI-F was strongly correlated with the domains of arousal, desire and satisfaction from the scale of FSFI (0.8<), but not the domains of lubrication, orgasm and pain, and also with domains of arousal, desire /frequency, desire /interest and pleasure from the scale of CSFQ, but not the domain of orgasm. In the divergent validity, the SIDI-F scores were not significantly correlated with the scores of marital adjustment test (MAT) both when all the women were included and when only women with HSDD were included. Furthermore, the questionnaire's reliability has been shown with excellent internal correlation (Cronbach's alpha = %90). Six items (receptivity, initiation, desire-frequency, desire-satisfaction, desire-distress and thought-positive) had a high overall correlation (r ≥0.7). Moreover, four items (relationship -sexual, affection, arousal -ease and arousal continuation) had a good overall correlation (r>0.5). Only one item (orgasm) had a week correlation (r = 0.1). In fact, all items of SIDI-F had the overall correlation of average to good except orgasm (17). 

Another study was done by Clayton et al. (2011) to assess the reliability and validity of SIDI-F as a tool to assess the intensity of HSDD. The population of this study comprised of 18-65 year old women with primary HSDD, female sexual arousal disease (FSAD) or without sexual function disorder (no FSD) in two non-therapeutic studies (in North America and Europe). In both studies, the average of the total SIDI-F score for women with HSDD was lower than that of women with FSD, indicating a proper discriminant validity of the instrument. Furthermore, in a study in North America, the average of the total SIDI-F score for women with HSDD was lower than that of the women with FSAD. In both studies, the total SIDI-F score had a high correlation with the total score of FSFI and CSFQ -F in women with HSDD, indicating convergent validity. In both studies, SIDI-F total score did not have a high correlation (0.02 and 0.23 for both studies) with the total score of MAT. The test-retest reliability of both studies has been displayed. For everyone, the ICC coefficient for the total score of SIDI-F with a 28-day internal was 85% and 90% in the studies of North America and Europe, respectively. Moreover, an appropriate internal coefficient was obtained for both studies in a way that the Cranach's alpha reported for studies of North America and Europe were 90% and 93%, respectively (25).

One of the advantages of our study was that to actualize face validity and content validity. We used both the quantitative and qualitative methods. Each of the quantitative and qualitative approaches was presented differently, but it was a complement to the study's perspective. We enjoyed the advantages of simultaneous or sequential use of different methods and assessed the study tool with a more exhaustive approach by combining different methods of validity assessment.

## Limitations

Researchers should be aware of the limitation based on culture for the data collected through this questionnaire; therefore, conducting more studies with larger sample size is highly recommended. Another limitation was lack of factor analysis. This study was conducted to assess the psychometric properties of the original version. Conducting a large study is suggested to assess the factor analysis of the Persian version of the SIDI-F. 

## Conclusion

The Persian version of the SIDI-F seems to be valid and reliable. Therefore, it can be used to identify women with low sexual desire in researches and sexual health programs provided by health centers in Iran to design appropriate interventions.

The Persian version of SIDI-F was developed through translation and cultural adaptation of the original SIDI-F. The findings revealed that despite minor cultural differences, the SIDI-F has acceptable validity and reliability and is easy to use. 
